# About stagnation and the emperor’s new clothes

**DOI:** 10.1186/1129-2377-16-3

**Published:** 2015-02-19

**Authors:** Geert Crombez

**Affiliations:** Department of Experimental-Clinical and Health Psychology, Ghent University, Henri Dunantlaan 2, 9000 Gent, Belgium

**Keywords:** Pain, Questionnaire, Stagnation, Psychology, Emotion, Headache

## Abstract

Innamorati and colleagues (Innamorati *et al.*, J Headache Pain 16:2, 2015) validated the “stagnation scale” and proposed to use it to screen for psychopathology. I have some critical comments to consider about the theoretical and clinical value of the instrument. First, items of the scale are not specific, and may equally well measure worry, self-worth and distress. Second, questionnaires are tools to assess the experiences of patients, but not hypothesized causal processes. The scale can thus not identify patients who repress emotions. Third, the routine use of this instrument will detract clinicians from what really is at stake in patients and what may help them.

## Correspondence

I read with interest the paper of Innamorati and colleagues [1]. The authors report the validation of a self-report instrument to screen for a “stagnation syndrome”, portrayed as a syndrome with a pedigree in traditional Chinese Medicine. The stagnation syndrome is characterized by a cluster of mind/body obstruction symptoms, such as ‘feeling that something is obstructed in the throat, or stuck in the chest and stomach’. The underlying cause of the syndrome is proposed as a repression of emotions which may lead in the long term to dysfunctions in body and mind.

The authors propose to use their instrument to screen for psychopathology and identify patients who respond poorly to standard headache management. The authors have performed sophisticated statistical analyses to demonstrate the construct validity of the instrument. The analyses are scholarly, and the results of the confirmatory factor analyses are outstanding. However, I do not share their enthusiasm. Unfortunately the measurement of stagnation is fundamentally flawed, and use of the instrument will detract clinicians from what really is at stake for patients and what may help these patients.

First, the psychometric validation of a self-report questionnaire is a complex enterprise, and requires several steps. Often neglected, but quintessential to the goal of measurement is the content validity of the instrument: Do the items of the questionnaire really reflect what is presumed to be measured? How do patients understand the items? Many of the items of this measure do not reflect stagnation (e.g. “I fear losing what I possess”, “I still miss the things I have already lost”), but simply capture the ruminations, worries and distress that are typically found in patients suffering from chronic pain. There is no need to come up with a new label. Research on worries and beliefs in patients is well-validated, and the assessment of worries and beliefs about illness and treatment, flows naturally into interventions that takes the complaints of patients seriously [2, 3].

Second, self-report questionnaires are excellent instruments to assess the phenomenological experience of patients. Beware of instruments that promise to do more, and allow conclusions about underlying causes. We have previously argued that somatization scales do not measure somatization [4]. Also here, one may hastily conclude that patients scoring high on the stagnation scale suffer from body/mind obstruction owing to a repressing of emotions. The instrument does not enable such conclusion. There are many alternative explanations that cannot be ruled out. Some of these alternative explanations are more relevant than the repression hypothesis. The instrument may simply measure the devastating consequences of any form of pain instead of the presumed hypothesis. Or, the instrument may simple measure physical symptoms of a more complex disease instead of a psychological mechanism.

Third, a routine use of the instrument to screen psychopathology in patients may unduly lead to a psychopathological view on pain. It may result in a triage of patients into a group with psychological problems and a group with “real” pain. Such dichotomization has proven unhelpful for patients. The routinely use of the instrument may also instigate a simplistic focus upon psychological interventions to target the repression of emotions. This limits the arsenal of psychotherapeutic interventions that are available and have proven to be effective.

In sum, stagnation and its measurement appear to be the emperor’s new clothes. One may believe that something new and exciting has been discovered, but its measurement is on shaky grounds, and evidence for it value is circumstantial. There are far better clothes for the emperor, well-validated and with a strong foundation in biopsychosocial theories of symptom perception and illness behavior. The psychology of pain has moved on [5].
